# Use of Local Melatonin with Xenogeneic Bone Graft to Treat Critical-Size Bone Defects in Rats with Osteoporosis: A Randomized Study

**DOI:** 10.3390/jfb15050124

**Published:** 2024-05-13

**Authors:** Karen Laurene Dalla Costa, Letícia Furtado Abreu, Camila Barreto Tolomei, Rachel Gomes Eleutério, Rosanna Basting, Gabriela Balbinot, Fabrício Mezzomo Collares, Pedro Lopes, Nelio Veiga, Gustavo Vicentis Oliveira Fernandes, Daiane Cristina Peruzzo

**Affiliations:** 1 Department of Periodontics, São Leopoldo Mandic Research Institute, Campinas 13045-755, SP, Brazil; 2 Laboratory of Dental Materials, Faculty of Dentistry of the Federal University of Rio Grande do Sul (UFRGS), Porto Alegre 90035-004, RS, Brazil; 3 Faculty of Dental Medicine, Universidade Católica Portuguesa, 3504-505 Viseu, Portugal; 4 Centre for Interdisciplinary Research in Health (CIIS), Faculty of Dental Medicine, Universidade Católica Portuguesa, 3504-505 Viseu, Portugal; 5 Missouri School of Dentistry & Oral Health, A. T. Still University, St. Louis, MO 63104, USA

**Keywords:** critical-size defects, bone mineral density, melatonin, bone regeneration, in vivo study

## Abstract

The aim of this study was to evaluate the effect of local administration of melatonin (MLT) on molecular biomarkers and calvaria bone critical defects in female rats with or without osteoporosis, associated or not with a xenogeneic biomaterial. Forty-eight female rats were randomly divided into two groups: (O) ovariectomized and (S) placebo groups. After 45 days of osteoporosis induction, two critical-size defects (5 mm diameter) were created on the calvaria. The groups were subdivided according to the following treatment: (C) Clot, MLT, MLT associated with Bio-Oss^®^ (MLTBO), and Bio-Oss^®^ (BO). After 45 days, the defect samples were collected and processed for microtomography, histomorphometry, and biomolecular analysis (Col-I, BMP-2, and OPN). All animals had one femur harvested to confirm the osteoporosis. Microtomography analysis demonstrated a bone mineral density reduction in the O group. Regarding bone healing, the S group presented greater filling of the defects than the O group; however, in the O group, the defects treated with MLT showed higher mineral filling than the other treatments. There was no difference between the treatments performed in the S group (*p* = 0.05). Otherwise, O-MLT had neoformed bone higher than in the other groups (*p* = 0.05). The groups that did not receive biomaterial demonstrated lower levels of Col-I secretion; S-MLT and S-MLTBO presented higher levels of OPN, while O-C presented statistically lower results (*p* < 0.05); O-BO showed greater BMP-2 secretion (*p* < 0.05). In the presence of ovariectomy-induced osteoporosis, MLT treatment increased the newly formed bone area, regulated the inflammatory response, and increased OPN expression.

## 1. Introduction

Bone tissue undergoes constant remodeling and renewal. This process happens due to the action of hormones, cytokines, and mechanical actions that the body undergoes daily [1]. An imbalance in bone remodeling can lead to osteoporosis, characterized by the fragility of bone architecture and greater susceptibility to fractures. Osteoporosis is a common pathological condition in perimenopausal women, as the decrease in estrogen production is related to greater bone resorption activity, and this physiological situation occurs at the same time as there is a decrease in the physiological synthesis of melatonin [2,3].

Melatonin (MLT) is a hormone related to the control of the circadian rhythm. Still, it also has activity in other functions of the human body, such as regulating body and bone mass [4]. Scientific information exists on melatonin’s activity on osteogenesis, through its activity in bone matrix production and mineralization, as well as through its antioxidant activity and anti-inflammatory control [5].

Factors involved in inflammation are directly linked with those critical factors for bone physiology and remodeling [6,7]. For example, pro-inflammatory cytokines (critical mediators of inflammatory responses) have been found to regulate bone metabolism, even in individuals without immunological diseases [8]. One of the most important of these cytokines is interleukin (IL)-6, which is produced by osteoblasts, monocytes, and T-cells and has been implicated in the pathogenesis of various metabolic bone diseases, including postmenopausal osteoporosis [9].

Thus, the role of MLT in bone metabolism has been investigated over the years by its activity in the secretion of osteoid matrix proteins such as type I Collagen (Col-I) [10,11], osteopontin (OPN) [12], osteocalcin (OCN) [11,13], and alkaline phosphatase (ALP) [14]; as well as growth factors such as bone morphogenetic proteins (BMPs) [4,15]. Animal research has demonstrated that systemically applied MLT has activity in healing bone wounds [16,17].

In dentistry, studies evaluated the topical application of MLT and its effectiveness through clinical and tomographic parameters, showing promising results at the implant osseointegration in fresh sockets and at the clinical stability of these peri-implant tissues [18,19]. A systematic review showed the use of melatonin was considered a reliable and feasible option as an adjunctive to the classical non-surgical periodontal treatment, with a significative improvement in the periodontal parameters (probing depth, clinical attachment level, bleeding on probing, plaque index, and gingival index), a significative reduction in the pro-inflammatory proteins (IL-1b, IL-6, and TNF-α), and better response for other biomarkers [20]. However, most information about biomolecular parameters came from cell culture research [21,22].

Systemically applied MLT could be an important aid in the process of bone neoformation because it directly suppresses osteoclast differentiation through the downregulation of the NF-κB pathway and subsequent NFATc1 transcription factor induction [23]. Otherwise, applying MLT locally, especially in situations of reduced oxygen tension, such as in osteoporosis cases, deserves more investigation. Despite the promising use of MLT to stimulate bone formation, there is a lack of scientific evidence, especially in situations that systematically affect bone metabolism, such as osteoporosis. Thus, the aim of the present study was to evaluate through microtomography, histomorphometry, and biomolecular analysis (Col-I, BMP-2, and OPN) the effect of local administration of MLT (associated with or not a xenogeneic biomaterial) applied to critical-size bone defects in female rats with or without osteoporosis.

## 2. Materials and Methods

The study was conducted and reported according to the ARRIVE guidelines [24], ISO 10993-6/2016 [25], and in compliance with the US National Research Council’s Guide for the Care and Use of Laboratory Animals, the US Public Health Service’s Policy on Humane Care and Use of Laboratory Animals, and the Guide for the Care and Use of Laboratory Animals. Before starting, the local ethical committee for animal research approved this study (n. 2019/005; 11 April 2019).

### 2.1. Sample Size Calculation

The sample size calculation was performed using ClinCalc.com, an online statistical calculator, to compare the results between two averages of a selected variable [26] in the same period (45 days). Previous results for newly formed bone area showed that group 1 (autogenous group) had 33.19% ± 10.7% of the percentual area, whereas group 2 (biomaterial, Bio-Oss^®^) had 11.90% ± 4.3%. A power of 90% with a significance level of 5% was established. The total sample size calculated was five defects per subgroup. Considering possible dropouts, we included one more animal/subgroup. Thus, a total sample size of 48 animals was reached to obtain a significant result.

### 2.2. Groups and Subgroups

The present study’s sample consisted of 48 Rattus norvegicus albinus, Wistar lineage, acquired from Biotério Anilab Ltd. (Paulínia, São Paulo, Brazil). All experimental procedures were carried out in the Vivarium of the São Leopoldo Mandic School of Dentistry in Campinas (SP, Brazil). The animals were equally divided into the ovariectomized group (O) (n = 24) and the placebo sham surgery group (S) (n = 24). Then, they were randomly subdivided (using dice, from 1 to 4 [following the order presented below]) into subgroups to receive the following treatments: blood clot (C), Bio-Oss^®^ (BO), melatonin (MLT), and MLT associated with Bio-Oss^®^ (MLTBO).

O-C and S-C: critical bone defect filled only with the blood clot; O-BO and S-BO: critical bone defect filled with 3.0 mg of xenogeneic biomaterial Bio-Oss^®^ (Geistlich, Switzerland); O-MLT and S-MLT: critical bone defect filled with 3.0 mg of pure melatonin powder (purity ≥ 96%); 4. O-MLTBO and S-MLTBO: critical bone defect filled with 1.5 mg of xenogeneic biomaterial Bio-Oss^®^ (Geistlich, Switzerland) associated with 1.5 mg of pure melatonin powder (purity ≥ 96%); the biomaterial was mixed before being implanted, with the use of saline solution. All groups received a collagen membrane (Bio-Gide^®^, Geistlich, Switzerland) after filling the defects, which was carefully placed covering the surgical sites.

### 2.3. Experimental Design

**Figure 1:**
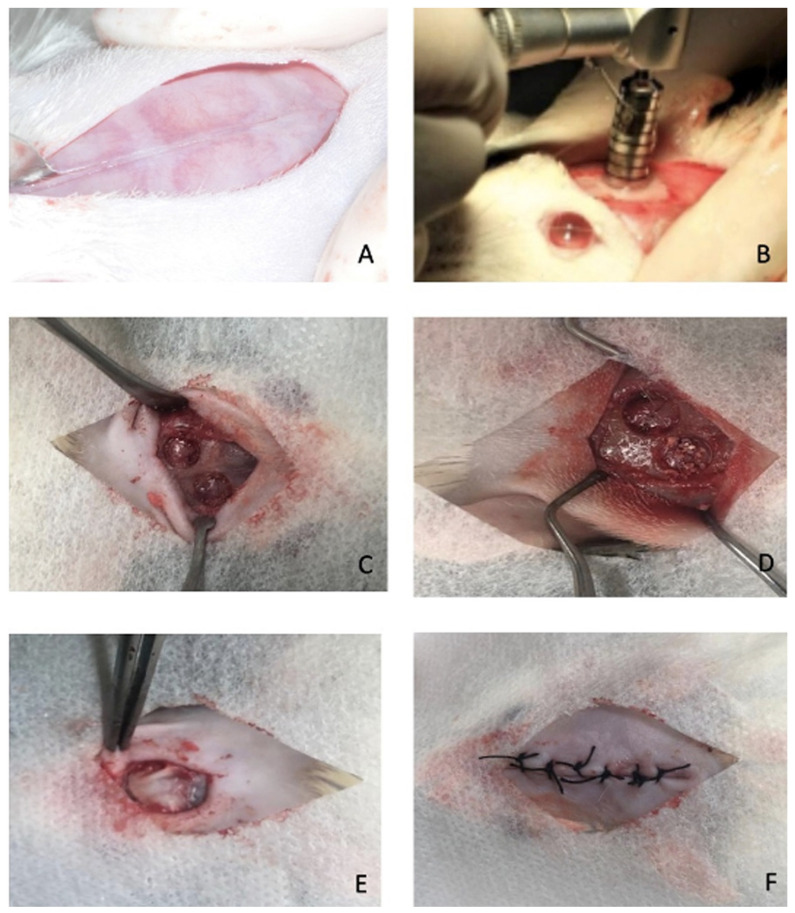
The surgical procedure was performed in the rats. (**A**) After the trichotomy procedure, a linear incision was performed; (**B**,**C**) two critical-size defects (5 mm); (**D**) biomaterial insertion to fill the defect; (**E**) membrane in position covering the defects; (**F**) suture performed.

After 45 days of osteoporosis induction by ovariectomy [27], the surgical procedures in the calvaria bone were started by performing intraperitoneal anesthesia with Xylazine (Dopaser^®^, Hertape Calier S.A., Juatuba, MG, Brazil) and Ketamine (Ketamine^®^ Agener, Agener União, Embu-Guaçu, SP, Brazil), in a respective proportion of 10:75 mg/kg. Trichotomy and asepsis were performed in the surgical region, using iodinated alcohol as an antiseptic at the incision sites. The soft tissue was raised, and two parasagittal bone defects were created, produced with a 5 mm trephine drill at low speed (400 RPM), with copious irrigation with saline solution (Figure 1A,B). Immediately after creating bone defects, animals from the O and S groups were randomly subdivided into subgroups to receive the following treatments: 1. O-C and S-C: critical bone defect filled only with the clot generated during surgery; 2. O-BO and S-BO: critical bone defect filled with 3.0 mg of xenogeneic biomaterial Bio-Oss^®^ (Geistlich, Switzerland); 3. O-MLT and S-MLT: critical bone defect filled with 3.0 mg of pure melatonin powder (purity ≥ 96%); 4. O-MLTBO and S-MLTBO: critical bone defect filled with 1.5 mg of xenogeneic biomaterial Bio-Oss^®^ (Geistlich, Switzerland) associated with 1.5 mg of pure melatonin powder (purity ≥ 96%). In all groups, after filling the defects, a collagen membrane (Bio-Gide^®^, Geistlich, Switzerland) was carefully placed covering the surgical sites. The flap was delicately repositioned and sutured (Figure 1C–F).

### 2.4. Euthanasia and Sample Collection

After 45 days, the animals were euthanized with deepening anesthetic, using 150 mg/kg of Sodium Thiopental Barbiturate and 10 mg/mL of Lidocaine intraperitoneally. The calvaria was removed and stored in 10% formaldehyde for micro-CT and histomorphometric analysis. For the histomorphometric analysis, 4 µm cross-section slices were made to obtain the histological slides. On the same day as the euthanasia, the tissue formed in the previously created and filled bone defects was removed with a Lucas curette, placed in vials, identified by group, and immediately stored at −80 °C for subsequent ELISA and PCR methods. Also, all animals had their right femur collected, stored in 10% formaldehyde, and evaluated by micro-CT to analyze the bone mineral density.

### 2.5. Micro-Computed Tomography (Micro-CT) Analysis

The samples were evaluated by a blind and calibrated researcher using high-resolution X-ray micro-CT (SMX-90 CT, ShimadzuCorp., Kyoto, Japan). Measurements were performed using imaging software (ImageJ v.1.54b, National Institutes of Health, Bethesda, MD, USA).

A standard area in the cortical portion of the bone was used to select a region of interest (ROI), and a color threshold was applied to segment different gray values in the images. This sample was used as a model to calculate the gray intensity of the materials in the analysis herein. Based on these, the threshold was set at 150 to 180. Bone area, bone volume, and the percentage of bone in the defects were measured on the stack of images obtained in the analysis.

The 48 femur samples were prepared and scanned via micro-CT (SMX-90 CT, ShimadzuCorp., Kyoto, Japan), with 360° images, and reconstructed on the inspeXioSMX-90CT (Shimadzu Corp., Kyoto, Japan), totaling 300 images per sample. Quantification and evaluation measurements of bone neoformation were performed using the ImageJ software (ImageJ; National Institutes of Health, Bethesda, MD, USA). An intraclass correlation coefficient test was performed on all specimens before analysis.

### 2.6. Histomorphometric Analysis

The collected specimens were prepared, with microtomies in the longitudinal direction at 4 µm, and stained using Masson’s Trichomic Stain method. A blind and calibrated researcher analyzed the histological slides histomorphometrically, considering the following parameters: total amount of neoformed bone and presence of residual Bio-Oss^®^.

The AxioVision software (v. 4.8.2, Carl Zeiss Microscopy, White Plains, NY, USA) was used for image acquisition. The images of the bone defect samples were acquired using a 20× flat objective lens and a 10× apochromatic objective lens at a fluorescence microscope (Axioskop 2 Plus, Zeiss, GmbH, Göttingen, Germany), equipped with a digital camera—a 10 MP digital camera with Aptina color CMOS sensor (MU1000, Zeiss, GmbH, Dresden, Germany). The bone defect was located on the slides and photographed in its entirety.

The slides were analyzed histomorphometrically, according to the total amount of newly formed bone and the presence of residual biomaterial parameters measured in the area (mm^2^). The acquired images were analyzed using ImageJ software (ImageJ^®^, National Institute of Health, Bethesda, MD, USA). For this analysis, the software’s area counting tool was used and calibrated with a micrometer scale in the image acquired from the samples at 200× magnification to measure both retro-cited parameters. For the analysis of bone neoformation, bone defects were located, and the area was measured from the stump of the bone defect.

### 2.7. Immunoassay for COL-I, BMP-2, and OPN

Col-I, BMP2, and OPN were quantified by osteoblastic cells cultured under different conditions using enzyme-linked immunosorbent assays (ELISA). The tissue collected from the bone wounds was homogenized, and the supernatant was collected and centrifuged at 5000× *g* for 15 min at 4 °C. ELISA was used to test aliquots of each sample to determine Col-I, BMP-2, and OPN levels according to the manufacturer’s recommendations (R&D Systems, Inc., Minneapolis, MN, USA).

The ELISA reaction was completed by adding 50 μL 2N sulfuric acid (H_2_SO_4_) to the substrate solution in each well. The absorbance was measured using a spectrophotometer (Epoch, Biotek, Winooski, VT, USA) at a wavelength of 450 nm. Col-I, BMP-2, and OPN levels were determined in pg/mL. All experiments were performed in triplicate.

### 2.8. Statistical Analysis

For the micro-CT data evaluation, normality assessment via the Shapiro–Wilk test was applied. A two-way ANOVA with post-hoc Tukey test was applied for bone density, percentage of neoformed bone, Tb.N, Tb.Th, and BV/TV data, while the Kurskall–Wallis test was applied for Conn.D and Tb.Sp data. The non-parametric Friedman test was used for histomorphometric data evaluation, followed by Dunn’s test for multiple comparisons between groups. ANOVA with Sidak and Bonferroni multiple comparison tests were applied to evaluate the biomolecular data. In all tests, the significance level was 5%.

## 3. Results

**Figure 2:**
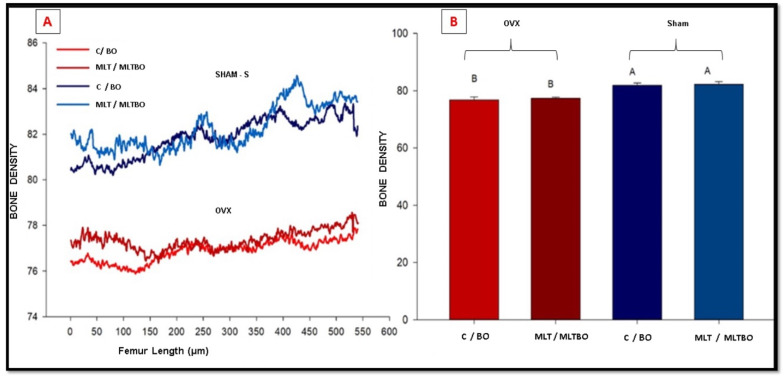
A line graph (**A**) and a column graph (**B**) demonstrate bone mineral density along the femurs of rats in the S and O groups, as determined via micro-CT analysis. Different letters indicate statistical differences between treatments.

Throughout the trans- and postoperative period, there were no complications related to the ovariectomies and bone defect procedures. There was no animal loss. Then, 48 samples of calvaria (96 defects) and 48 femurs were analyzed. Micro-CT analysis of the animals’ femurs demonstrated that ovariectomy surgery led to low mineral bone density, compatible with osteoporosis (Figure 2).

**Figure 3:**
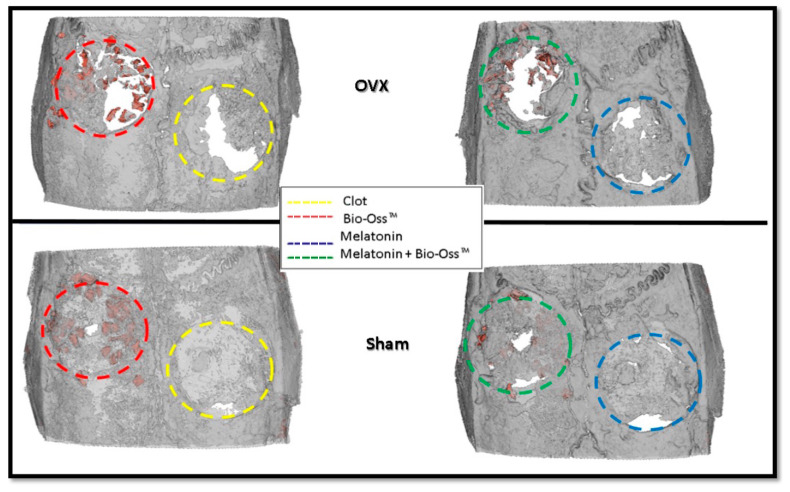
Three-dimensional calvaria reconstruction representing groups S and O and their respective defect treatments: Yellow: Clot (C); Red: Bio-Oss (BO); Green: Melatonin (MLT); Blue: Melatonin + Bio-Oss (MLTBO).

To reconstruct a 3D image of the calvaria, four samples (one from each treatment) were randomly chosen: two from the S group and two from the O group (Figure 3). Concerning the healing in the extension of the bone defect, the S group generally presented greater filling of the bone defects than the O group. However, it is worth highlighting that, even in the O group, the bone defects that were filled with MLT presented visually greater filling than the other groups.

**Figure 4:**
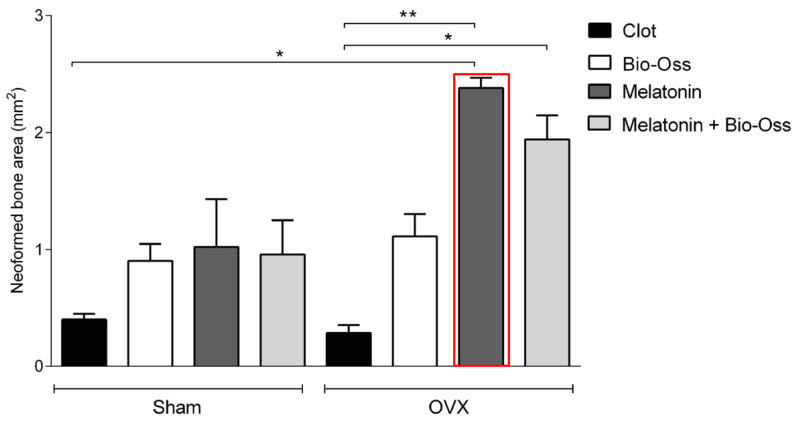
Histomorphometrical analysis and comparison between the S and O groups. The red square shows a statistically significant difference between the MLT and other treatments. There was a significantly greater response (*/**) from the MLT treatment in group O compared to the MLT treatment in group S. The significance level established was 5%. (* = *p* < 0.05; ** = *p* < 0.01)

**Figure 5:**
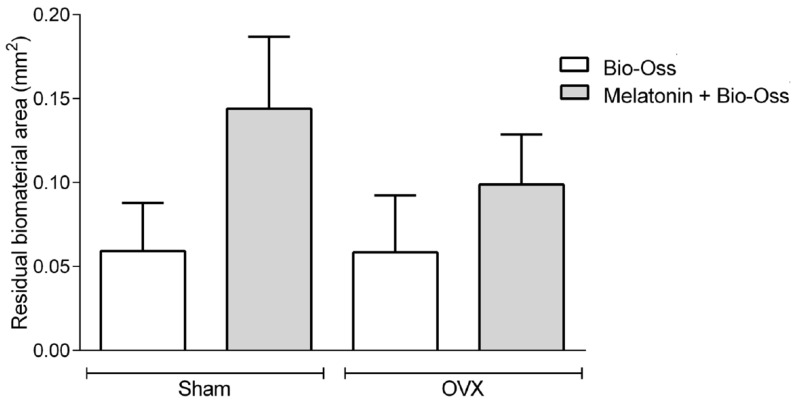
Column graph showing the area of residual biomaterial. There was no statistical difference between groups and treatments.

Histomorphometrically, in the O-MLT group, the results demonstrated that the area of newly formed bone tissue was significantly greater than the other treatments (*p* < 0.05); however, there was no difference between MLT and MLT-BO treatments. In the S group, no statistically significant differences were observed between treatments. Intergroup analysis identified differences between O-S and MLT-OVX, with no differences for the other treatments (Figure 4). The treatments significantly influenced the total measurement of the amount of neoformed bone tissue and the presence of residual biomaterial (Figure 5). The area of neoformed bone tissue was greater in BO, MLT, and MLTBO treatments. In the O group, a greater area of neoformed bone was observed in the MLT and MLTBO treatments. The results for C and BO treatments were similar in both S and O groups. In MLTBO or pure MLT treatments, the result was significantly greater for the area of neoformed bone tissue. MLT co-administered with BO allows it to dissociate and be well distributed throughout the tissues, with no apparent inflammatory tissue circulating. In C treatment, scar tissue with inflammatory areas and a clot undergoing resorption were apparent. Figure 5 shows data regarding the presence of residual particles of the biomaterial in the S-BO, S-MLTBO, O-BO, and O-MLTBO groups. No statistically significant differences exist within or between groups (*p* > 0.05).

**Figure 6:**
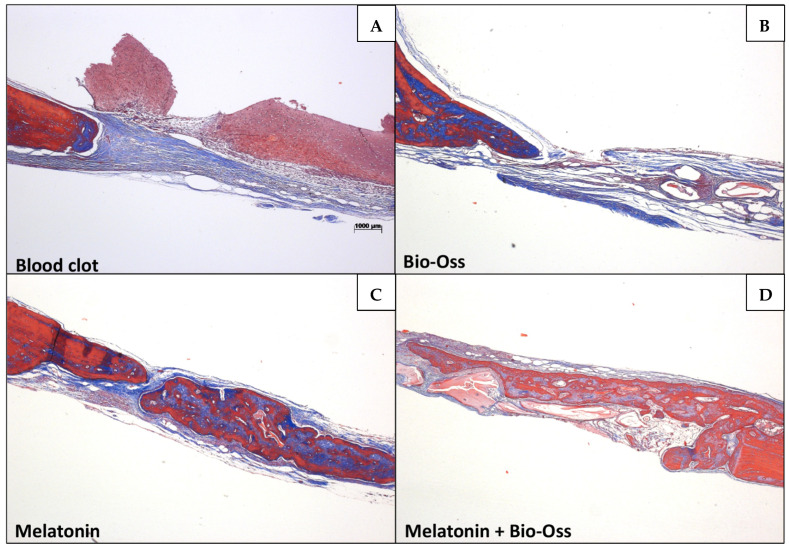
Sham Group (40× magnification, Masson’s Trichomic staining). (**A**) = C (blood clot group); (**B**) = BO (Bio-Oss^®^ group); (**C**) = MLT (melatonin group); (**D**) = MLTBO (melatonin with Bio-Oss^®^ group).

**Figure 7:**
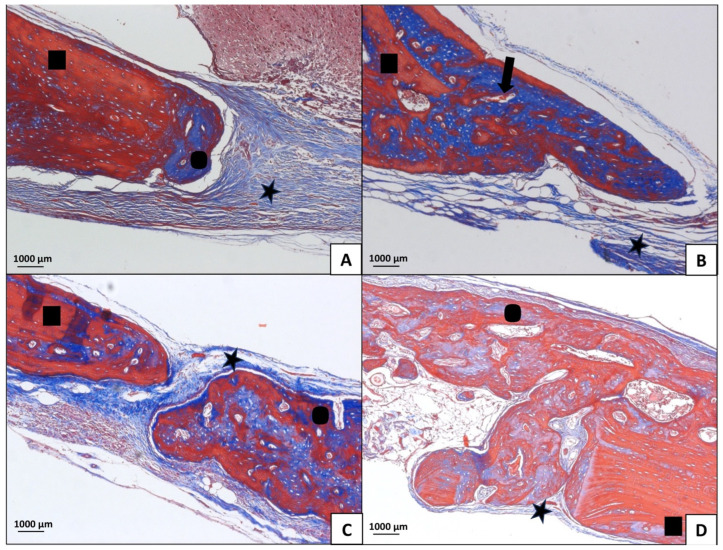
Sham Group (100× magnification, Masson’s Trichomic staining). (**A**) = C (blood clot group); (**B**) = BO (Bio-Oss^®^ group); (**C**) = MLT (melatonin group); (**D**) = MLTBO (melatonin with Bio-Oss^®^ group). The star symbol corresponds to fibrous connective tissue rich in collagen fibers; residual Bio-Oss^®^ is represented by arrows; the bone edges are made up of newly formed bone tissue, represented by the circle; squares represent mature (old) bone.

**Figure 8:**
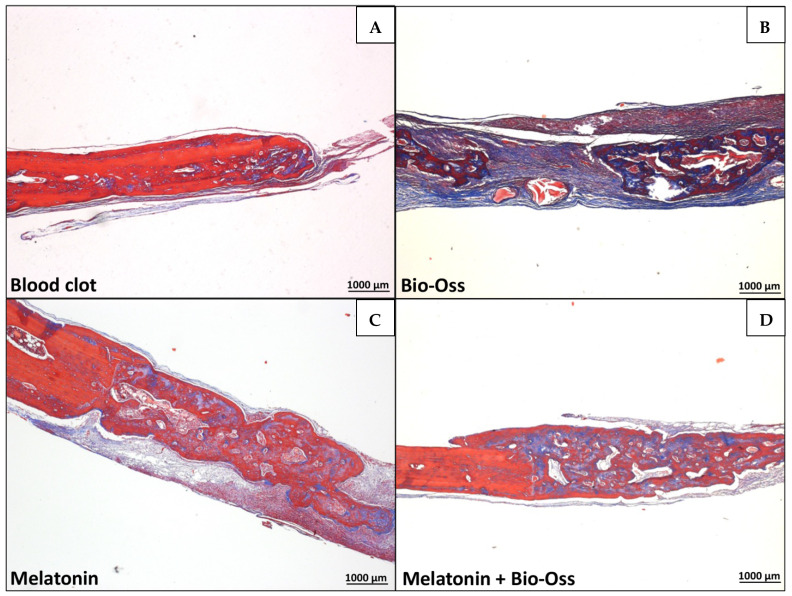
Ovariectomized Group (40× magnification, Masson’s Trichomic staining). (**A**) = C (blood clot group); (**B**) = BO (Bio-Oss^®^ group); (**C**) = MLT (melatonin group); (**D**) = MLTBO (melatonin with Bio-Oss^®^ group).

**Figure 9:**
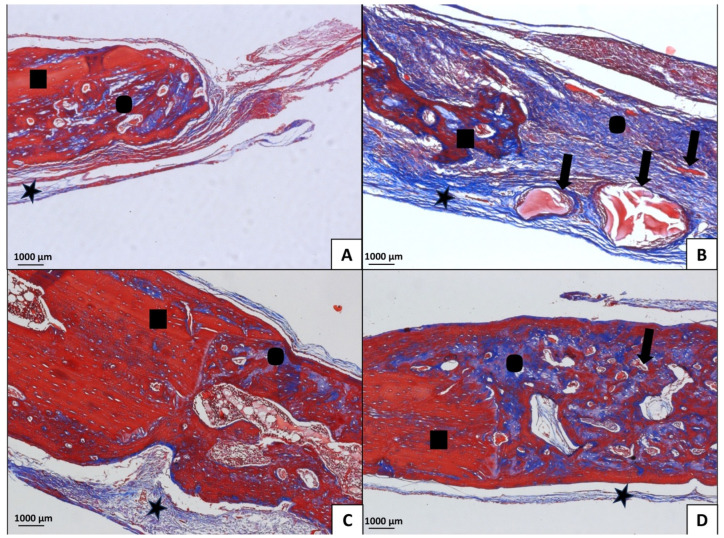
Ovariectomized Group (100× magnification, Masson’s Trichomic staining). (**A**) = C (blood clot group); (**B**) = BO (Bio-Oss^®^ group); (**C**) = MLT (melatonin group); (**D**) = MLTBO (melatonin with Bio-Oss^®^ group). The star symbol corresponds to fibrous connective tissue rich in collagen fibers; residual Bio-Oss^®^ is represented by arrows; the bone edges are made up of newly formed bone tissue, represented by the circle; mature (old) bone is represented by squares.

In the S-C, S-BO, O-C, and O-BO groups, there was a predominance of trabecular bone, with very evident areas of resorption and few collagen fibers. The MLT treatment induced a visually larger area of newly formed bone compared to the other treatments. Comparing BO, MLT, and MLTBO treatments, a larger area of neoformed bone was noted compared to the C treatment. A significantly larger area of neoformed bone tissue was observed when MLT was used alone, without co-administration. MLT treatment was significantly superior for the O group compared to the S group (Figure 6, Figure 7, Figure 8 and Figure 9). A larger area of inflammatory infiltrate was observed only around the clots in the C and BO subgroups, probably due to the presence of both Bio-Oss^®^ and Bio-Gide^®^ biomaterials and the presence of MLT. In specimens from the MLT treatment, fewer osteoclasts can be distinguished. In the MLT treatment, bone neoformation can be observed, as evidenced by new cells both at the site of the defect and at a distance, as well as the presence of mature cells with a lamellar bone tissue matrix.

**Figure 10:**
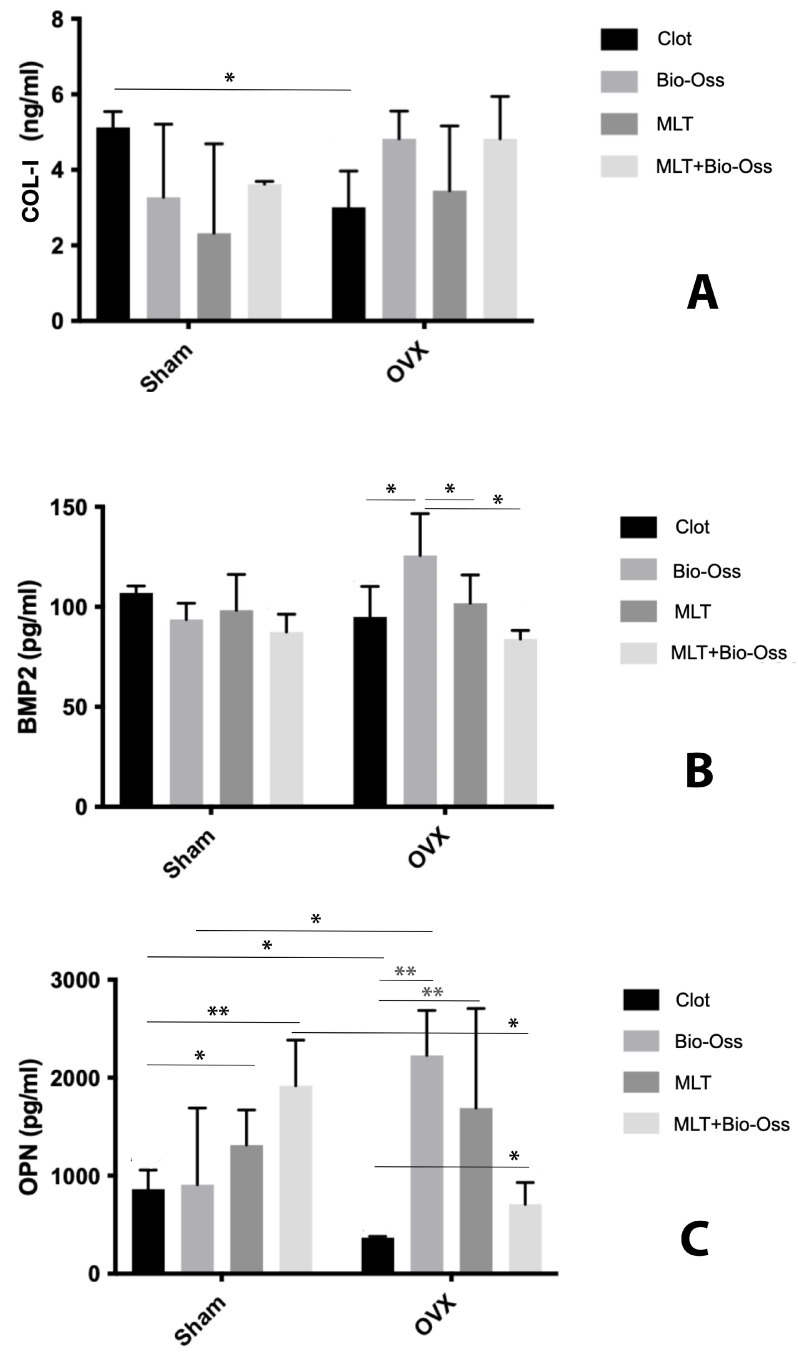
(**A**) Col-I; (**B**) BMP-2; (**C**) OPN analysis. * (*p* < 0.05); ** (*p* < 0.01).

For biomolecular assessments, the results of Col-I analysis in intragroup comparisons demonstrated no difference in protein secretion between groups (Figure 10A). Multiple comparisons for the intergroup analysis revealed no significant difference between the groups, except for the O-C group, which showed lower protein secretion (Figure 10A). For the BMP-2 production, the O-BO group showed significantly higher results than the O-C, O-MLT, and O-MLTBO groups and no difference was observed between the S-C, S-BO, S-MLT, and S-MLTBO groups (Figure 10B). In the intergroup comparison, no significant differences were observed between systemic conditions for any treatment used (Figure 10B). For the OPN production, the intragroup evaluation demonstrated that the S-MLT and S-MLTBO groups presented significantly higher results than the S-C group. The S-BO group demonstrated OPN production like the other groups. The O-BO, O-MLT, and O-MLTBO groups presented results that did not differ from each other, and the O-C group presented significantly lower OPN production than the other groups (Figure 10C). In intergroup comparisons, the S-C and S-MLTBO groups presented greater OPN production than the O-C and O-MLTBO groups. Still, the O-BO group presented greater OPN production than the S-BO group, while the S-MLT and O-MLT groups showed no differences between them (Figure 10C).

## 4. Discussion

Osteoporosis is directly related to bone activities, resulting in reduced osteoblast activity and continued osteoclast cell activity. It is common to find patients with osteoporosis taking medications, such as bisphosphonates. Within this aspect, Rebelo et al. [28] demonstrated the influence of bisphosphonates on implant failure; the authors concluded a high mean failure rate of implant osseointegration (49.96%) was found to be associated with the medication, regardless of the bisphosphonates’ generation; therefore, the failure rate was lower in patients using second-generation bisphosphonates (Alendronate and Pamidronate) and higher with the IV administration compared to the oral administration. Then, strategies were studied in order to improve the development of functional biomaterials for delivering multiple growth factors, which are effective strategies for bone repair in a temporally controllable carrier and, thus, improve the therapeutic effect [29].

Several studies have demonstrated that MLT has an important role in stimulating the activity of bone neoformation, regeneration, and remodeling, ref. [30] especially in individuals with osteoporosis [4,11,21,31,32]. Also, MLT was a reliable and feasible option as an adjunctive to the classical non-surgical periodontal treatment, improving the periodontal parameters and significantly causing a reduction of the pro-inflammatory cytokines (IL-1b, IL-6, and TNF-α) [20]. However, despite positive clinical outcomes, there is little information regarding the microtomographic, histomorphometric, and biomolecular patterns of bone neoformation associated with MLT, especially in cases where there is systemic involvement.

Within these premises, the present study evaluated, through histomorphometric, microtomographic, and biomolecular analysis, the effect of the local application of MLT on critical bone defects in order to mimic experimental clinical situations studied in humans [4,30,33], comparing with the use of xenogeneic bone substitute (Bio-Oss^®^), as well as the combination of these treatments.

Different MLT administration routes and different forms of application have been studied. Among studies in animal models, local application of the lyophilized hormone is observed [10,16,18,33], systemic administration via intraperitoneal routes [11,17], and the use of the hormone via gavage [13,34]. In the present study, the local application of MLT was chosen to mimic procedures performed clinically, such as alveolar preservation procedures [35] and implant installation [36]. Additionally, as tested herein, the local application of MLT has the positive point of not interfering with other systems in the organism [31] compared with its systemic administration.

Animal models of osteoporosis are tools for studying ways to treat and prevent osteoporosis [27,37]. Various methods in animal models have been used to induce osteoporosis, with different study designs and periods between the osteoporosis-induction procedures and the confirmation of osteoporosis. Previous studies demonstrated the induction of osteoporosis after ovariectomy of Wistar rats under different periods: 7–11 weeks [38], 80 days [39], 8–12 weeks [40], and 60 days [41]. In the present study, surgical procedures on the calvaria were done 45 days after ovariectomy to ensure the development of osteoporosis, which was confirmed via microtomography analyses of the rats’ femurs, showing a significant reduction in bone mineral density in the group of rats that underwent ovariectomy.

It has been demonstrated that MLT acts as a local growth factor with a paracrine effect [10]. Its direct action on osteoblasts induced a higher rate of pre-osteoblast maturation in quantity and speed, with a higher rate of bone matrix production and subsequent calcification [12,21,42]. In the present study, the histomorphometric evaluation demonstrated that only the O-MLT group presented significant results with greater bone formation than the other treatments. This result may be based on the MLT osteogenic and regenerative potential in cellular hypoxia situations [43] and osteoporosis, as previously documented in peri-implant areas [30]. It is worth presenting that newly formed woven primary bone (immature) is primarily deposited in the boundary of the bone defect, with numerous newly differentiated osteoblasts and developing bone matrix (osteoid matrix), with a high proportion of osteocytes appearing on the bone and expanding to the central region of the defect, keeping a centripetal pattern, leading to the progressive (according to the period) confluence of bone trabeculae [44].

The present finding regarding the greater area of bone neoformation in group O deserves focus due to the strong tendency observed in the literature to use the MLT hormone as an adjunct in bone-repair processes, especially in patients with chronic dysfunctions, such as osteoporosis patients [11,21,31,32].

The molecular mechanisms that lead to bone repair continue to be studied. One of the most accepted hypotheses refers to the recruitment of osteoclastic cells of hematopoietic origin in patients whose state of cellular hypoxia is evident, as in cases of reduced bone mineral density [30]. Another hypothesis is based on the MLT’s favorable action in alkaline phosphatase (ALP) activity; thus, MLT would favor the increase in BMP-2 and osteoprotegerin (OPN) levels, leading to an increase in bone mineral density and bone neoformation [32], as well as to OPN and Col-I gene expression and protein secretion [12].

At the study’s microtomography and histomorphometry analyses, MLT alone presented results similar to other treatments in the percentage of neoformed bone and bone density parameters, independent of systemic conditions. These results corroborate with other findings, suggesting that MLT may be involved in optimizing bone neoformation and inhibiting resorption mediated by osteoclasts, neutralizing reactive oxygen and nitrogen species [33]. Such inferences may support the findings of the present study, as MLT would act locally by reducing the expression of RANK by osteoblasts and RANK-L by osteoclasts while increasing the production and secretion of proteins related to the bone-healing process (e.g., Type-I Collagen, BMP, osteopontin, and osteocalcin). It was also observed that although the application of MLT, alone or in combination, did not result in a significant increase in Col-I production, the group that did not receive biomaterial in the condition of induced osteoporosis (O-C group) exhibited statistically lower results for protein secretion. It can be inferred that using biomaterials, including MLT, may be recommended in cases of low bone density as an alternative to stimulate bone neoformation.

Observing the OPN protein production, this research demonstrated that treatments carried out with local application of a biomaterial obtained similar results in both the S and the O groups. The results demonstrated a difference in the O-C group. These results demonstrated that using a biomaterial in osteoporosis conditions could help in OPN secretion, an important protein in bone neoformation. Another interesting assessment is the similarity in OPN secretion in the O group, as the results from the O-BO group demonstrated similar concentrations to those from the O-MLT and O-MLTBO groups. The results demonstrated in the O group may suggest that MLT used locally as a biomaterial could assist the process of bone neoformation, as has been pointed out by other studies [12,13].

Regarding BMPs, BMP-2 and BMP-7 have received more attention regarding bone formation. Both promoted bone healing. Results using rhBMP-7 associated with a thin nanostructure HA-coated implant promoted a greater new bone area than the same implants without rhBMP-7 [45]. Our study assessed BMP-2 production; the results showed that the O-BO group had significantly greater BMP-2 secretion than the other groups. This result could be justified by the commercial biomaterial’s stability within the surgical wound as a reference osteoconductive biomaterial, allowing the establishment of the osteoblastic cell network. Compared to cell culture research [46], the results of this research were divergent because cell culture research demonstrated biomaterial action at early osteogenesis. The present study also demonstrated that the local use of MLT as a biomaterial induced similar BMP-2 concentrations in S and O group animals, as demonstrated in S-MLT and O-MLT groups, as well as in S-MLTBO and O-MLTBO groups. Moreover, the present findings could demonstrate that the stimuli that appear at the beginning of osteogenesis were still noticeable in an advanced phase of bone formation, as shown in the graphs.

A limitation of the present study is that it uses only one euthanasia period (45-day period). We hypothesize that at a 45-day period, the control group that received only the clot may have been favored by the natural healing time of the bone defect without the induction promoted by xenogeneic biomaterial or MLT. Therefore, from a future perspective, an analysis with an intermediate euthanasia time with an increase in the sample number could be justified.

## 5. Conclusions

The results of the present study demonstrated that MLT improves bone repair in osteoporotic tissue. This article justifies that new studies should be carried out to validate such findings before clinical application.

## Data Availability

All data were inserted in the article.
